# Neuromorphic Computing of Optoelectronic Artificial BFCO/AZO Heterostructure Memristors Synapses

**DOI:** 10.3390/nano14070583

**Published:** 2024-03-27

**Authors:** Zhao-Yuan Fan, Zhenhua Tang, Jun-Lin Fang, Yan-Ping Jiang, Qiu-Xiang Liu, Xin-Gui Tang, Yi-Chun Zhou, Ju Gao

**Affiliations:** 1 School of Physics and Optoelectric Engineering, Guangdong University of Technology, Guangzhou Higher Education Mega Center, Guangzhou 510006, China; 2112115054@mail2.gdut.edu.cn (Z.-Y.F.);; 2 School of Advanced Materials and Nanotechnology, Xidian University, Xi’an 710126, China; 3 Department of Physics, The University of Hong Kong, Hong Kong 999077, China

**Keywords:** BFCO/AZO, optoelectronic, artificial synapse, neuromorphic computing

## Abstract

Compared with purely electrical neuromorphic devices, those stimulated by optical signals have gained increasing attention due to their realistic sensory simulation. In this work, an optoelectronic neuromorphic device based on a photoelectric memristor with a Bi_2_FeCrO_6_/Al-doped ZnO (BFCO/AZO) heterostructure is fabricated that can respond to both electrical and optical signals and successfully simulate a variety of synaptic behaviors, such as STP, LTP, and PPF. In addition, the photomemory mechanism was identified by analyzing the energy band structures of AZO and BFCO. A convolutional neural network (CNN) architecture for pattern classification at the Mixed National Institute of Standards and Technology (MNIST) was used and improved the recognition accuracy of the MNIST and Fashion-MNIST datasets to 95.21% and 74.19%, respectively, by implementing an improved stochastic adaptive algorithm. These results provide a feasible approach for future implementation of optoelectronic synapses.

## 1. Introduction

With the bottleneck of the von Neumann architecture, the rapid expansion of data and information places increasing demands on the efficiency of computers to capture and manipulate [1,2,3]. Neuromorphic technologies, which imitate the structure of the human brain, have garnered attention recently. In the human brain, 10^11^ neurons are connected by 10^14^ synapses to form a neural network that is capable of effectively computing vast amounts of data [4]. These neuromorphic devices can increase power consumption and running speed by using analog processing to perform vector matrix multiplication, the fundamental calculation of machine learning [5]. Consequently, the integration of amnesia devices into artificial synapses is considered a crucial advancement in the development of neuromorphic computing hardware [6,7]. This technology has the potential to execute intricate and advanced mathematical calculations on a large scale, with exceptional speed and efficiency. Additionally, it can address the limitations posed by the von Neumann computer architecture [8].

Various materials, such as metal oxides [9,10], perovskites [11,12,13], and organic materials [14,15], have been used to fabricate artificial synapses based on memory resistors. Studies of synaptic behavior using memristors have usually focused on simulating a variety of manifestations of synaptic plasticity. Neurons generate postsynaptic currents (PSC) in response to external stimuli, which are used for signal delivery and storage. The synaptic weight, also known as the value of the PSC, reflects the strength of the connection between two neurons. Changes in synaptic weight can be categorized into short-term plasticity (STP) and long-term plasticity (LTP) based on duration [16]. Further research has explored multiple plasticities of artificial synaptic devices, including paired-pulse facilitation (PPF) [17] and spike-timing-dependent plasticity (STDP) [18].

Compared to purely electrically triggered devices, neuromorphic devices for photonic stimulation provide a non-contact writing method that may be more conducive to increased processing speed [19,20]. For example, Shrivastava et al. successfully fabricated a light-stimulated synaptic memristor (LSSM) based on ZnO/Zn_2_SnO_4_ heterostructure with reversibly tunable conductance state properties. The synaptic characteristics of this fully photonically controlled memory synapse were revealed by the enhancement and inhibition behavior of violet and red light pulse stimulation, respectively [21]. Yu et al. prepared a novel optoelectronic neuromorphic device based on pn junction-modified oxide thin-film transistors, which responds to broadband visual data in the neuromorphic system from the ultraviolet to the visible region [22].

In this work, based on a pn heterojunction (p-BFCO/n-AZO) with a bilayer structure, an optoelectronic artificial synaptic Au/BFCO/AZO/FTO device is fabricated. The device exhibits favorable electrosynaptic properties and effectively replicates the behaviors of biological synapses, including PSC, STP, LTP, PPF, and STDP. In addition, the plasticity of the device is enhanced by light pulses from a violet light source, which induce holes that change the conductivity state of the device by removing electrons from interfacial oxygen vacancies (V_O_^s^). And the number of the applied light pulses is increased to simulate the synaptic transition from short-term memory (STM) to long-term memory (LTM). Finally, the convolutional neural networks (CNNs) constructed with Au/BFCO/AZO/FTO artificial synapses achieve recognition accuracies of 95.21% and 74.19% for the MNIST and Fashion–MNIST datasets, respectively, by implementing an improved stochastic adaptive algorithm. A photostimulated synaptic memristor with reversible controllability based on BFCO/AZO heterostructure is prepared to provide a new research idea for the development of photoelectric synapses.

## 2. Materials and Methods

First, the substrate was cleaned using acetone, absolute ethanol, and deionized water for 5 min via ultrasonic means. The target material required for magnetron sputtering was an AZO (ZnO:Al_2_O_3_ = 98:2 wt%, 99.99% purity) target. The experimental parameters were as follows: sputtering time 20 min, RF power 80 W, Ar gas flow rate 55 sccm, pressure 0.5 Pa [23,24]. The films were then annealed in air at 600 °C for 15 min. The BFCO precursor solutions were then prepared using the sol-gel method by dissolving Bi(NO_3_)_3_·5H_2_O and Fe(NO_3_)_3_·9H_2_O in 2-Methoxyethanol, respectively, and first stirred at room temperature for 10 min. Bi(NO_3_)_3_·5H_2_O was then dissolved in glacial acetic acid and stirred continuously for 20 min at a temperature of 50 °C. The above solutions were mixed with each other and stirred for 30 min. Then, acetylacetone was added as the stabilizer and stirred for 30 min to obtain the precursor solution. The AZO films were ultrasonically cleaned with anhydrous ethanol for 5 min and then rotary coated with BFCO film at 3500 rpm. The wet film was dried on a heated platform at 200 °C for 5 min and then annealed at 400 °C for 10 min. In order to reach the appropriate thickness, the previous procedure was repeated several times to deposit amorphous films. Subsequently, the samples were prepared by annealing at a temperature of 600 °C for 30 min. Finally, Au electrodes with 1 mm diameter were deposited by magnetron sputtering at room temperature. The AZO and BFCO samples were chemically analyzed using X-ray emission spectroscopy (XPS) (Thermo Scientific K-Alpha, Thermo Fisher Scientific, Waltham, MA, USA). The structural properties of the samples were evaluated using X-ray diffraction (XRD) techniques (Rigaku Ultima IV, Rigaku, Tokyo, Japan). Furthermore, the device’s artificial synaptic behavior was measured using a Keithley 2611 semiconductor analyzer (KI, Cleveland, OH, USA).

## 3. Results

**Figure 1:**
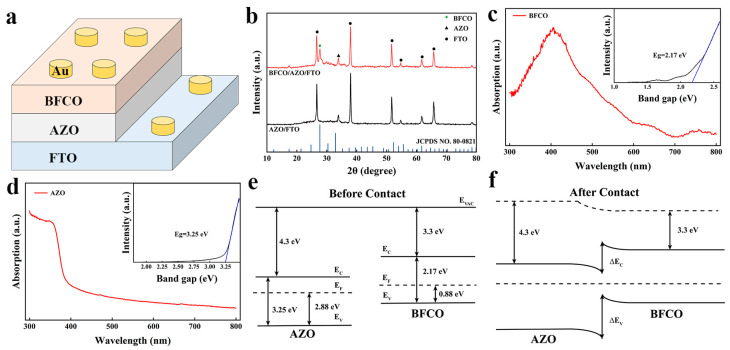
(**a**) Schematic of Au/BFCO/AZO/FTO memristor device structure. (**b**) X-ray diffraction (XRD) patterns of AZO and BFCO/AZO. (**c**) The absorption spectrum of BFCO (inset energy band gap of BFCO). (**d**) The absorption spectrum of AZO (inset energy band gap of AZO). Equilibrium band gap energy diagrams of AZO and BFCO (**e**) before and (**f**) after contact.

The schematic structure of the Au/BFCO/AZO/FTO device is shown in Figure 1a. The X-ray diffraction (XRD) patterns of the AZO and BFCO/AZO samples are shown in Figure 1b, and a peak at 2θ = 28° is observed in the XRD test of the BFCO sample, which can be attributed to Bi_7_CrO_12.5_ (JCPDS No. 42-0527) or Bi_12_(Bi_0.5_Fe_0.5_)O_19.5_ (JCPDS No. 80-0821) phases. These phases are difficult to distinguish; however, Bi_7_CrO_12.5_ may be possible candidates, and they can usually be formed at higher doping concentrations and moderate membrane growth conditions, respectively [25]. Figure 1c,d show the absorption spectra of BFCO and AZO films, respectively, and show that the BFCO film absorbs more light at a wavelength of 405 nm. The inset shows the corresponding bandgap widths of the BFCO and AZO films, and it can be obtained that the bandgap widths of BFCO and AZO are 2.17 eV and 3.25 eV, respectively. Figure S1a,b show the valence band spectra of BFCO and AZO films, indicating that BFCO is a p-type semiconductor and AZO is an n-type semiconductor. Figure S1a,b show the XPS analysis of BFCO film and AZO film. Figure 1e shows the energy band diagrams of BFCO and AZO, BFCO has a lower electron affinity energy and narrower forbidden band width than AZO [26,27], and the two contact to form a staggered heterojunction [28], as shown in Figure 1f. This heterojunction generates a built-in electric field at the AZO/BFCO interface.

**Figure 2:**
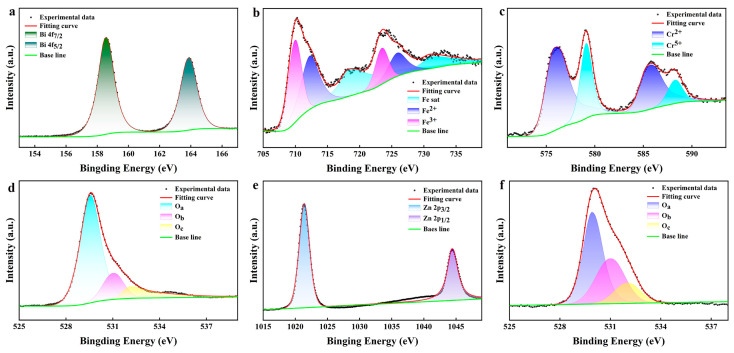
XPS spectra of BFCO film: (**a**) Bi 4f, (**b**) Fe 2p, (**c**) Cr 2p, and (**d**) O 1s. XPS spectra of AZO film: (**e**) Zn 4p and (**f**) O 1s.

In order to further analyze the composition of the samples, we performed XPS characterization of the BFCO and AZO films, respectively. The full XPS spectra of BFCO and AZO are shown in Figure S1c,d. The spectrum of BFCO film shows the characteristic peaks of C, O, Fe, Bi, and Cr, while the spectrum of AZO film shows the characteristic peaks of C, Zn, and O. Figure 2a portrays the 4f orbital XPS spectrum of Bi, depicting discernible peaks denoted as Bi 4f_7/2_ and Bi 4f_5/2_, exhibiting binding energies of 158.53 eV and 163.84 eV, respectively. Figure 2b exhibits the 2p orbital XPS spectrum of Fe, manifesting distinct Fe 2p_3/2_ and Fe 2p_1/2_ peaks. The asymmetry observed in these peaks intimates the presence of multiple valence states of Fe, where Fe^2+^ and Fe^3+^ are the prominent valence ions within the BFCO film. Figure 2c illustrates the 2p orbital in the Cr XPS spectrum, showcasing distinct Cr 2p_3/2_ and Cr 2p_1/2_ peaks. The asymmetry observed in these peaks suggests the coexistence of two distinct valence states for chromium: Cr^2+^ and Cr^5+^. Figure 2d shows the 1s orbital XPS spectra of O element in the BFCO thin films, and the O 1s spectra have three peaks at 529.56 eV, 531.0 eV, and 532.1 eV, where 529.56 eV and 532.1 eV are the lattice oxygen and surface-bound oxygen, respectively, and 531.0 eV, which is located in the middle of the two peaks, is the oxygen vacancy. Figure 2e shows the 2p orbital XPS spectrum of element Zn in AZO; the spectrum is divided into two peaks, Zn 2p_3/2_ and Zn 2p_1/2_, with binding energies of 1021.39 eV and 1044.37 eV, respectively. Figure 2f shows the 1s orbital XPS spectrum of element O in the AZO thin film, and similarly, the O 1s spectrum at 529.9 eV, 531.99 eV, and 532 eV has three peaks; it can be observed that the oxygen vacancy peaks of AZO are higher, and the presence of oxygen vacancies can make the film have a better resistive state change. These XPS results can provide evidence that the experimentally prepared BFCO films and AZO films are relatively pure.

**Figure 3:**
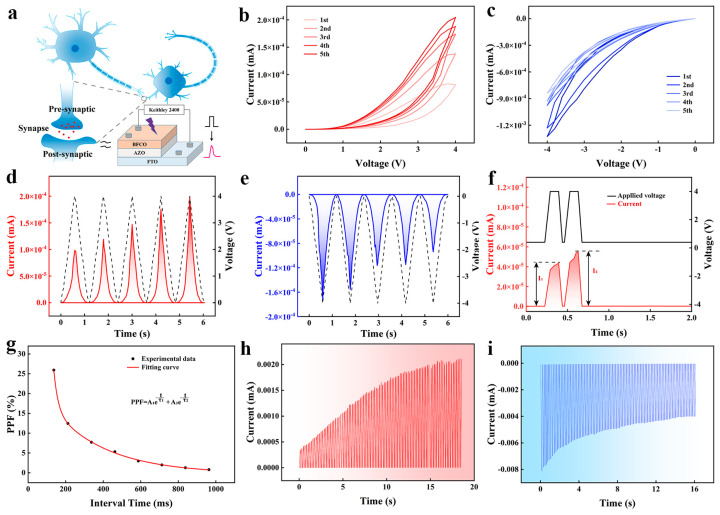
(**a**) Schematic diagram of synapse in the biological system and memristor device used as an artificial synapse. (**b**) I-V curve under the forward voltage bias sweep (0 to +4.0 V). (**c**) I-V curve under the reverse voltage bias sweep (0 to −4.0 V). (**d**) The STP effects of the device under triangular wave voltage stimulation. (**e**) The STD effects of the device under triangular wave voltage. (**f**) Paired pulse (4 V, 100 ms) triggers EPSC on the artificial synapse. (**g**) The relationship between PPF and interval time (ΔT). The LTP and LTD effects of the devices are shown in (**h**) and (**i**).

Figure 3a illustrates the analogy between a biological synapse and a memristor: the PSC through the device mimics the electrical impulse signals generated by a presynaptic neuron stimulating the top electrode. As shown in Figure 3b,c, the current of the device increases (decreases) continuously under ±4 V voltage sweep, and the current increases (decreases) less and less as the number of sweeps increases [29]. As shown in Figure 3d,e, the STP and STD characteristics of the device were evaluated under positive and negative triangle waves with an amplitude of 4.0 V. These are similar to the changes in synaptic weight under the spike signal in the real nervous system. When the conductance of the device is considered as the synaptic weight, the device conductance increase (decrease) can be considered as biological synaptic signal enhancement (inhibition) [30]. Furthermore, the device’s STP/STD characteristics are reproducible. Again, the channel corresponds to a conductance similar to the synaptic weight. Paired pulse facilitation (PPF) is a type of STP behavior that is associated with complex synaptic activity. In biological synapses, two consecutive excitatory stimuli to a presynaptic neuron increase the strength of the response of a postsynaptic neuron. Similarly, our artificial synaptic device can be used to simulate PPF behavior by applying two pulses with different time intervals, as shown in Figure 3f, thus detecting the response current (EPSC) of the device. The EPSC following the first and second peaks is denoted as I_1_ and I_2_, accordingly, and the PPF index is defined by the following equation:

(1) $$PPF=\frac{I_{2}-I_{1}}{I_{1}}\times 100\%$$

Figure 3g displays both the empirically determined PPF index and the PPF index curve. The relationship between the PPF index and ∆t can be modeled using the double exponential formula as follows:

(2) $$PPF=C_{1}\exp\left(-\frac{t}{\tau_{1}}\right)+C_{2}exp(-\frac{t}{\tau_{2}})$$

C_1_ and C_2_ are the initial facilitation of each phase, and the relaxation time constants τ_1_ and τ_2_ are the fast and slow decay terms of the function, respectively. For the PPF feature, τ_1_ and τ_2_ were fitted to 23.07 ms and 293.74 ms, respectively, which is consistent with the relaxation factor in biological synapses [31].

The transition from STP/STD to LTP/LTD can be accomplished by applying repetitive spikes to the device. LTP and LTD are the primary synaptic mechanisms involved in the process of learning. These mechanisms are replicated by gradually changing the conductance state of the Au/BFCO/AZO/FTO device. To realize the transition from STP to LTP, we applied 100 pulses (height ±4.0 V, width 100 ms) to the device. The current increases proportionally with the longer retention time as the number of pulses increases in Figure 3h. Similarly, as shown in Figure 3i, the LTD effect can be achieved by applying equal pulses of opposite amplitude. The device accomplishes electrical stimulation of synaptic activity by utilizing gradual variations in conductance, which can be employed for both synaptic enhancement and inhibition.

**Figure 4:**
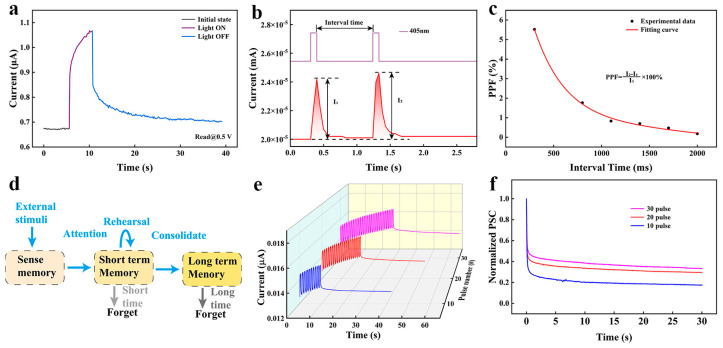
(**a**) Light-induced response characteristics of the device under a single light exposure. (**b**) Paired pulse (4 V, 100 ms) triggers EPSC on the artificial synapse. (**c**) The relationship between PPF and interval time (ΔT). (**d**) Schematic diagram of memory model of the human brain. (**e**) Excitation of EPSC with a fixed wavelength of 405 nm and different numbers of light pulses. (**f**) The STM to LTM transition was achieved by repetitive light pulse stimulation.

Synaptic plasticity in the nervous system is influenced by both electrical activity and external environmental inputs, particularly light signals [20,32]. Changes in action potentials generated in synapses can be dynamically regulated under different light intensities, enabling the regulation of synaptic weights through neuronal activity. Such changes that are dependent on light stimulation can be considered as light-induced plasticity. As shown in Figure 4a, this paper introduces the use of visible light at a 405 nm wavelength to modulate the device conductance and verify light-induced synaptic plasticity. The device’s PPF behavior under light was investigated by varying the time interval between two consecutive light pulses. As shown in Figure 4b, the current response under the second light stimulation is significantly larger than that of the first light stimulation. It can also be noticed that the current after the reaction is higher than the initial current before the stimulation and can last for a brief amount of time. As shown in Figure 4c, the exponent for light-induced PPF gradually decreases as the pulse interval increases. The fits for τ_1_ and τ_2_ are 2.54 ms and 316.89 ms, respectively, which are similar to those for the electrically induced PPF and biological synapse. As shown in Figure 4d, the mental memory and forgetting model of the human brain suggests that all information obtained by the sense organs from the surrounding world is stored in the human sensory system. Through repeated stimulation and reinforcement, short-term memory (STM) is transformed into long-term memory (LTM) [33,34]. We replicated the synaptic transition process from STM to LTM by increasing the quantity of light pulses administered when the device was placed in the dark. As shown in Figure 4e, the PSC increased from 0.0153 μA to 0.017 μA under photostimulation with 10, 20, and 30 pulses with a width of 0.5 s. In addition, it can be observed that at the end of the pulses, there was a decrease in the rate of current decay after the 30-pulse response. Figure 4f shows the memory retention curve during forgetting as a function of time after the stimulus. The curve was fitted using the Kohlrausch [35] stretch exponential function.

(3) $$I\left(t\right)=I_{0}exp[-{\left(\frac{t}{\tau }\right)}^{\beta }]$$

where I(t) is the PSC at the moment; I_0_ is the maximum PSC after stimulation; τ is the relaxation time constant; and β is the stretch index from 0 to 1. In the relaxation curves after 10, 20, and 30 spike stimulations, the τ of the device was 0.1678 s, 4.922 s, and 6.4572 s, respectively. In this scenario, the decay rate of I(t)/I_0_ is reduced following an augmentation in the quantity of light pulses, a phenomenon that can be described as memory preservation during forgetting.

**Figure 5:**
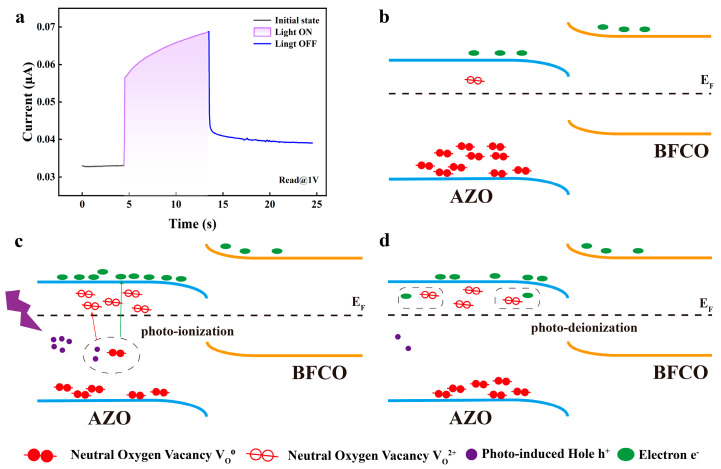
Schematic illustration of the working principle of photonic synapses: (**b**) Arrangement of electron–oxygen vacancy bands at the AZO/BFCO interface under thermal equilibrium conditions; (**c**) Light-induced hole production and ionization of oxygen vacancies; (**d**) Deionization of oxygen vacancies under dark environment.

The photomemory mechanism was identified by studying the energy band structures of AZO and BFCO. BFCO has a lower electron affinity and a narrower band gap compared to AZO. A staggered heterojunction band structure is generated when BFCO comes into contact with AZO. This heterojunction generates a built-in electric field at the BFCO/AZO interface. The breadth of the interfacial barrier is dependent on the concentration of charged V_O_^s^ and is of utmost importance in determining the behavior of memory switching [36]. Typically, in n-type semiconductor oxides, defects such as V_O_^s^ exhibit insulating α-type behavior in their neutral stable state (V_O_^0^) and β-type behavior in their substable charged states (V_O_^2+^). In our work, the change of the charged state of V_O_^s^ can be realized by trapping excited holes near the valence band maximum (VBM) under 405 nm violet excitation. A rational mechanism for the operation of photonic synapses is proposed, as shown in Figure 5a. Setting the device with a readout voltage of 0.5 V, Figure 5b shows the energy band arrangement at the AZO/BFCO interface under thermal equilibrium conditions. As shown in Figure 5c, after 405 nm violet illumination, the charged state of the V_O_^s^ can be realized by trapping excited holes near the valence band maximum (VBM). The ionization process can be represented by the following equation

(4) $$V_{o}^{0}+O_{\mathrm{WB}}^{2-}+2h^{+}\to \frac{V_{o}^{2+}}{O_{i}}+2e^{-}$$

Here, O_WB_^2−^ is weakly bound oxygen, and O_i_ is tissue oxygen. When the device is irradiated with violet light, then V_O_^0^ is photoionized into V_O_^2+^ in the AZO/BFCO cross section, leading to an increase in PSC. From the previous XPS test of AZO, it is known that in zinc oxide, the proportion of oxygen vacancies is large, which facilitates the excitation of more charged oxygen vacancies and electrons. At the end of the violet light irradiation, the oxygen vacancies undergo de-ionization, as shown in Figure 5d, and the charged oxygen vacancies and electrons in the device begin to decrease, leading to a decrease in PSC. However, a portion of the charged oxygen vacancies are retained, and it can be observed that the current remains greater than the initial current value for a certain period of time after illumination. The device’s current can be restored to its initial value by applying a negative voltage to accelerate oxygen vacancy deionization.

**Figure 6:**
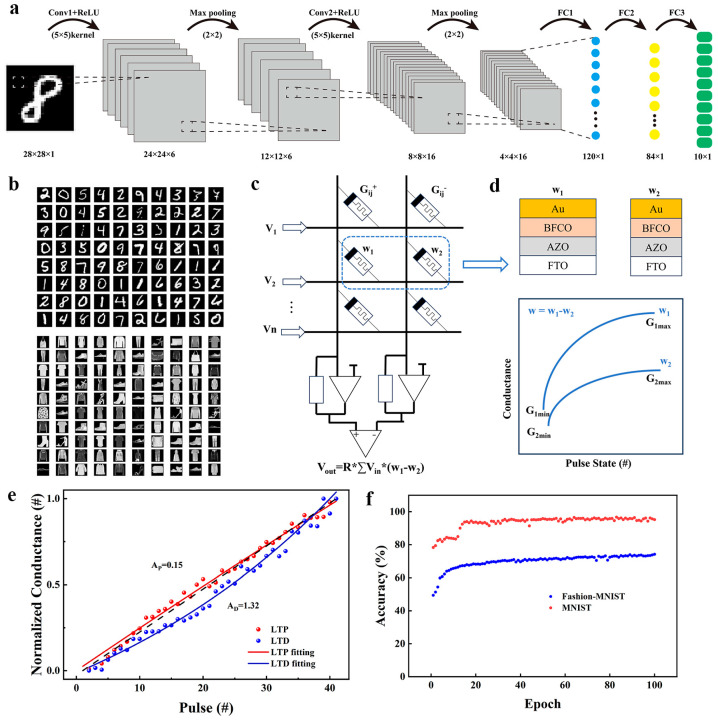
(**a**) The designed CNN architecture for MNIST dataset classification. (**b**) Training sample of the MNIST and the Fashion–MNIST. (**c**) The differential-pair framework of the memristor network. (**d**) The differential-pair of the improved weight update method. (**e**) Nonlinear relationship of normalized conductivity under 40-pulse stimulation. (**f**) Recognition accuracy of Au/BFCO/AZO/FTO memristor.

In order to explore the capabilities of our artificial synaptic device in machine learning applications, we constructed a convolutional neural network (CNN) structure using PyTorch [37]. We then utilized this network to classify two datasets: one for recognizing handwritten digits from the American National Standards and another for categorizing fashion clothing items. Figure 6a depicts the schematic of our network architecture, comprising two convolutional layers, each followed by a maximum pooling layer. The kernel width (k_w_), kernel height (k_h_), input channel (c_i_), and output channel (c_o_) of the first convolutional layer are k_w_ = k_h_ = 5, c_i_ = 1, and c_o_ = 6, respectively, while the second convolutional layer is k_w_ = k_h_ = 5, c_i_ = 6, and c_o_ = 16, respectively.

The three fully connected layers are then immediately used as outputs, and the final outputs are 10 dimensions, which we use as 0–9 identifiers for classification and finally achieve the accuracy of data classification. Figure 6b shows the two datasets, MNIST and Fashion–MNIST. Currently, the most common training method for memristor networks involves using a 1-transistor-1-memristor setup to create a differential pair, as shown in Figure 6c. This work utilizes an enhanced iteration of the random gradient descent algorithm (SGD) for training neural networks. The algorithm uses random adaptive techniques that involve identifying the positive and negative directions of each update and applying a constant value for the update. It removes the necessity of calculating precise pulse numbers and automatically adjusts and compensates for weights. Figure 6d illustrates a differential pair consisting of two memristors with various sizes, obtained by adjusting the size of the device. A collection of memristor differential pairs is created with unique conductivity values and changing rates.

A series of 40-cycle square wave pulses with an amplitude of ±4 V were applied to the device. The effect of nonlinear weights updating on the neural network during the long-term enhancement/decrease (LTP/D) process of the device was investigated [38]. The update behavior is modeled using the following equations:

(5) $$G_{LTP}=\beta \left(1-e^{-\frac{N}{\alpha }}\right)+G_{min}$$

(6) $$G_{LTD}=-\beta \left(1-e^{-\frac{N-N_{max}}{\alpha }}\right)+G_{max}$$

(7) $$\beta =\frac{G_{max}-G_{min}}{1-e^{\frac{-N_{max}}{\alpha }}}$$

(8) $$A_{P/D}=\frac{1.726}{\frac{\alpha }{N_{max}}+0.162}$$

where G_max_ and G_min_ represent the upper and lower bounds of the conductivity displayed by the device, respectively. Nmax denotes the number of intermediate conductances. The parameter α plays a key role in determining the nonlinear behavior of the weights update. β is a correlation function of α. In addition, A_P_ and A_D_ are utilized to represent the nonlinearity observed in the LTP and LTD curves, respectively. As shown in Figure 6e, the A_P_ and A_D_ of the Au/BFCO/AZO/FTO device are 0.15 and 1.32, respectively, indicating that the device has good linearity. As shown in Figure 6f, 100 trainings are performed on Au/BFCO/AZO/FTO devices, where the average accuracy of digit set training reaches more than 90% after 13 cycles and reaches 95.21% after 100 cycles of training. Even under garment set training, the correct recognition rate can reach more than 70% after 32 cycles of training, and the recognition accuracy reaches 74.19% after 100 cycles of training.

## 4. Conclusions

In summary, we have fabricated an optoelectronic artificial synapse based on BFCO/AZO heterostructure, which has excellent electrosynaptic properties. And the holes induced by the 405 nm violet light source can be used to change the conductivity state of the device by removing electrons from the interfacial oxygen vacancies (V_O_^s^), which makes the device exhibit photomemory blocking behavior and successfully mimic the transition of biological synapses from STM to LTM. Finally, the convolutional neural network (CNN) built using Au/BFCO/AZO/FTO artificial synapses achieved 95.21% and 74.19% recognition accuracy for MNIST and Fashion–MNIST datasets, respectively, by implementing an improved stochastic adaptive algorithm.

## Data Availability

The data that support the findings of this study are available within the article and its Supplementary Material.

## Supplementary Materials

The following supporting information can be downloaded at: https://www.mdpi.com/article/10.3390/nano14070583/s1, Figure S1. (a) XPS valence band spectra of BFCO film. (b) XPS valence band spectra of AZO film. (c) XPS analysis of BFCO film. (d) XPS analysis of AZO film.
