# Supplementary material for: Neuromorphic Computing of Optoelectronic Artificial BFCO/AZO Heterostructure Memristors Synapses
Source: Nanomaterials (Basel). 2024 Mar 27;14(7):583. doi: 10.3390/nano14070583 (PMC11013421; doi:10.3390/nano14070583)
Supplement: Supplementary file 1 [file nanomaterials-14-00583-s001.zip › nanomaterials-2915550-supplementary.pdf]

nanomaterials

Supplementary Materials

Neuromorphic Computing of Optoelectronic Artificial BFCO/AZO Heterostructure Memristors Synapses

Zhao-Yuan Fan [1], Zhenhua Tang [1,*], Jun-Lin Fang [1], Yan-Ping Jiang [1], Qiu-Xiang Liu [1], Xin-Gui Tang [1], Yi-Chun Zhou [2] and Ju Gao [3]

1 School of Physics and Optoelectric Engineering, Guangdong University of Technology, Guangzhou Higher Education Mega Center, Guangzhou 510006, China; 2112115054@mail2.gdut.edu.cn (Z.-Y.F.)
2 School of Advanced Materials and Nanotechnology, Xidian University, Xi'an 710126, China
3 Department of Physics, The University of Hong Kong, Hong Kong 999077, China
* Correspondence: tangzh@gdut.edu.cn

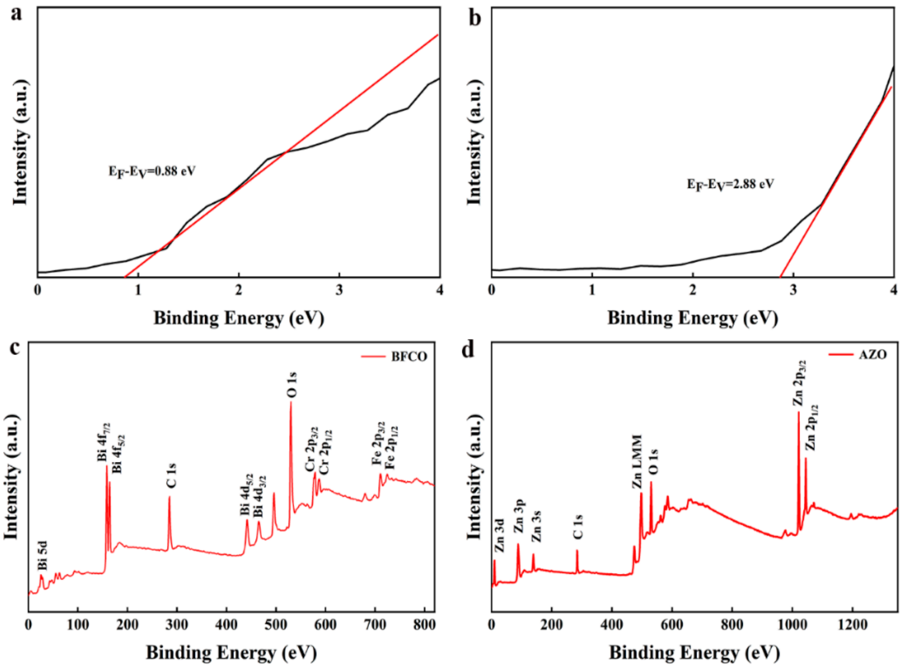

Figure S1. (a) XPS valence band spectra of BFCO film. (b) XPS valence band spectra of AZO film. (c) XPS analysis of BFCO film. (d) XPS analysis of AZO film.
